# Feeling Blue or Seeing Red? Similar Patterns of Emotion Associations With Colour Patches and Colour Terms

**DOI:** 10.1177/2041669520902484

**Published:** 2020-02-02

**Authors:** Domicele Jonauskaite, C. Alejandro Parraga, Michael Quiblier, Christine Mohr

**Affiliations:** Institute of Psychology, University of Lausanne, Switzerland; Computer Science Department, Computer Vision Center, Universitat Autònoma de Barcelona, Spain; Institute of Psychology, University of Lausanne, Switzerland

**Keywords:** colour, affect, semantic associations, metaphors, Geneva Emotion Wheel

## Abstract

For many, colours convey affective meaning. Popular opinion assumes that perception of colour is crucial to influence emotions. However, scientific studies test colour–emotion relationships by presenting colours as patches or terms. When using patches, researchers put great effort into colour presentation. When using terms, researchers have much less control over the colour participants think of. In this between-subjects study, we tested whether emotion associations with colour differ between terms and patches. Participants associated 20 emotion concepts, loading on valence, arousal, and power dimensions, with 12 colours presented as patches (*n* = 54) or terms (*n* = 78). We report high similarity in the pattern of associations of specific emotion concepts with terms and patches (*r* = .82), for all colours except *purple* (*r* = .−23). We also observed differences for *black*, which is associated with more negative emotions and of higher intensity when presented as a term than a patch. Terms and patches differed little in terms of valence, arousal, and power dimensions. Thus, results from studies on colour–emotion relationships using colour terms or patches should be largely comparable. It is possible that emotions are associated with colour concepts rather than particular perceptions or words of colour.

## Introduction

Across languages and cultural traditions, we use colour to express and convey emotional states. We *feel blue*, *see red,* or we are *green with envy*; we wear white to weddings and black to funerals; and we give red hearts to our loved ones on Valentine’s Day. It seems that colour–emotion associations are ubiquitous (e.g., Adams & Osgood, 1973; Allen & Guilford, 1936; Hupka, Zaleski, Otto, Reidl, & Tarabrina, 1997; Ou et al., 2018; Valdez & Mehrabian, 1994). One detail should, however, not be neglected: The first examples are concerned with affective colour expressions, omnipresent in language (Soriano & Valenzuela, 2009), while the remaining examples are concerned with colour perceptions. Actual research into colour–emotion associations have used both colour patches (e.g., Valdez & Mehrabian, 1994) and colour terms (e.g., Adams & Osgood, 1973). When using patches, great effort is put into how colours appear by controlling colour presentation (see Valdez & Mehrabian, 1994). Researchers can carefully control the three colour dimensions of hue, saturation, and lightness (Hunt & Pointer, 2011) and test a myriad of colours. When using terms, researchers have little control over the colour participants think of. In such studies, researchers can present fewer colours and the colours are presented as words. Considering these methodological differences, we have little a priori knowledge to predict whether and, if so, which colour–emotion relationships would be the same or different, when a person is presented with patches or terms.

The roles of language versus perception have been considered in various theoretical frameworks. The conceptual metaphor theory emphasises the role of language in colour–emotion associations (Lakoff & Johnson, 1999). This theory suggests that abstract concepts like affect (i.e., emotions, mood, evaluations, preferences) are metaphorically, or metonymically, linked to more concrete perceptual experiences such as colour (see also Meier & Robinson, 2005; Soriano & Valenzuela, 2009). This link would help people to better understand and describe their affective experiences. Meier and Robinson (2005) used this framework to explain the omnipresent association between positivity and lightness, which manifests in metaphorical expressions like *bright day* and *dark thoughts* (Adams & Osgood, 1973; Lakens, Fockenberg, Lemmens, Ham, & Midden, 2013; Meier, Robinson, Crawford, & Ahlvers, 2007; Specker et al., 2018). Meier and Robinson (2005) additionally argued that such metaphorical associations further reinforce these links across time, through language and cultural learning. At some point, the metaphors might function independently from the original perceptual associations and so dissociate language and perception (e.g., there is no clear perceptual base for expressions like *green with envy*, *feeling blue*, and *yellow-bellied*).

Other frameworks are less readily apt to explain colour–emotion associations through affective metaphors. If we take the example of *yellow*^1^ and *joy*, this association is widely spread (Burkitt & Sheppard, 2014; Dael, Perseguers, Marchand, Antonietti, & Mohr, 2016; Jonauskaite, Althaus, Dael, Dan-Glauser, & Mohr, 2019; Kaya & Epps, 2004; Lindborg & Friberg, 2015; Sutton & Altarriba, 2016) but has no equivalent metaphorical expression, at least not in English, German, French, Lithuanian, Dutch, or Spanish. Rather, yellow is metaphorically associated with negative emotions in different languages (e.g., *yellow-bellied*—to be cowardly or easily scared; *Gelb vor Neid sein*—to be envious, *rire jaune—*forced laughter to hide embarrassment). Similarly, despite the expression *feeling blue* signifying *sadness* in English, *blue* has been repeatedly associated with positive emotions (Adams & Osgood, 1973; Jonauskaite & Mohr, 2019; Kaya & Epps, 2004; Valdez & Mehrabian, 1994; Wexner, 1954) and is generally a liked colour (Eysenck, 1941; Jonauskaite, Dael, et al., 2019; Palmer & Schloss, 2010) in English- and non-English-speaking countries. In such cases, visual colour perception may play a more important role than language. These associations might be rooted in repeated perceptual associations between a colour and an emotional situation, such as feeling joyful when the sun is shining or feeling good when looking at clear blue water. Such propositions have been made to explain colour preferences (Ecological Valence Theory, Palmer & Schloss, 2010) and various cross-modal relationships (structural or statistical correspondence, C. Spence, 2011).

When appreciating the implications of the various frameworks, we would have to expect that certain colour–emotion associations might be more strongly reinforced by the linguistic system and colour metaphors (e.g., the associations between *blue*, and between *sadness* and *yellow* and *negative emotions*) and others through the perceptual system (e.g., the associations between *yellow*, and between *joy* and *blue* and *positive emotions*). In the former case, the actual colour presentation might play a more important role, while in the latter case, colour presentation might play a less important role. Moreover, there might be colour–emotion associations reinforced through both systems. For instance, the metaphorical expression *seeing red* associates *red* with feelings of *anger*, which would indicate a linguistic influence. Nonetheless, when one gets angry, blood rushes to the face (Benitez-Quiroz, Srinivasan, & Martinez, 2018), and so the perception of red faces in an angry situation might further strengthen the association between *red* and *anger*.

One possible approach for investigating which system, perceptual or linguistic, reinforces colour–emotion associations to a greater extent is to compare emotion associations with colour presented as patches (i.e., perceptual stimuli) versus as terms (i.e., linguistic stimuli). Despite the large body of empirical studies on colour–emotion associations, few studies have compared these methods directly (see Wang, Shu, & Mo, 2014 for a notable exception). More commonly, researchers worked separately with either colour patches (Allen & Guilford, 1936; D’Andrade & Egan, 1974; Fugate & Franco, 2019; Hanada, 2018; Kaya & Epps, 2004; Manav, 2007; Palmer, Schloss, Xu, & Prado-Leon, 2013; Valdez & Mehrabian, 1994) or colour terms (Adams & Osgood, 1973; Hupka et al., 1997; Soriano & Valenzuela, 2009; Sutton & Altarriba, 2016). Researchers working with perceptual colour stimuli have criticised research studies that used linguistic colour stimuli on the basis of vagueness and imprecision (e.g., Fugate & Franco, 2019; Valdez & Mehrabian, 1994). In other words, when presenting linguistic stimuli, unlike when presenting perceptual stimuli, one does not know the exact colour (how light, how saturated) participants visualised. Thus, it is unclear if emotions are attached to particular physical properties of colours, particular colour terms, or instead to particular colour concepts (i.e., abstract representations of colour combining colour perceptions with colour terms).

Furthermore, different methodologies of colour assessment might tap into different associative mechanisms. For instance, Wang et al. (2014) studied which colour–emotion associations are *natural* (i.e., arise due to perceptual pairing) and which are *social* (i.e., arise due to linguistic and cultural pairing) in Chinese participants. They tested implicit valence associations with colour terms and colour patches of *red* and *blue*. The authors demonstrated that *red* was evaluated both positively and negatively when presented as a patch. When *red* was presented as a term, it was evaluated exclusively positively. The authors suggested that the association between *red* and negative emotions (e.g., *anger*) is natural. Thus, it is present when a *red* patch is perceived. When *red* is treated linguistically, however, such pairing may be overshadowed by the exclusively positive connotations of *red* in Chinese culture (i.e., good fortune, success, beauty, joy, etc.; Toulson, 2013)—the social associations. Hence, to obtain a more complete picture, emotion associations should be tested with both colour terms and patches.

In this study, we investigated the extent to which colour–emotion associations are comparable between colour patches and colour terms by asking participants to associate as many or as few emotion concepts (Geneva Emotion Wheel [GEW]; Scherer, 2005; Scherer, Shuman, Fontaine, & Soriano, 2013) with 12 colours. The emotion concepts differentially loaded on the emotion dimensions of *valence* (positive–negative), *arousal* (high arousal–low arousal), and *power* (strong–weak; Fontaine, Scherer, Roesch, & Ellsworth, 2007). Thus, we were able to analyse colour associations with specific emotions and with emotion dimensions. We also tested emotion intensity. Crucially, one group of participants associated emotion concepts with colour terms (Experiment 1), while the other group associated emotion concepts with colour patches (Experiment 2). We chose the 11 basic colour terms in French plus *turquoise* for the term condition and focal colours that best matched each term for the patch condition (Lindsey & Brown, 2014). The focal colours are representative members of colour categories (Abbott, Griffiths, & Regier, 2016), thus participants were likely to imagine focal colours when presented with colour terms. We expected some degree of dissimilarity between colour terms and colour patches, especially for colours that might have divergent meanings as a term and as a patch (e.g., *blue*, *yellow*).

## Method

### Participants

We recruited 132 first-year university students (23 males) with a mean age of 20.52 years (95% confidence interval [CI] = [20.21, 20.82]). Seventy-eight participants (15 males) took part in Experiment 1 (*M_age_* = 21.19, 95% CI_age_ = [20.88, 21.51], range: 19–24) and a new group of 54 participants (8 males) took part in Experiment 2 (*M*_age_ = 19.60, 95% CI_age_ = [19.10, 20.01], range: 18–26). An independent samples *t* test showed that participants in Experiment 1 (*M* = 21.19, standard deviation = 1.40) were slightly older than in Experiment 2 (*M* = 19.56, standard deviation = 1.66), *t*(130) = 6.13, *p* < .001, *d* = 1.06. This difference occurred because participants in Experiment 1 participated at the end of the academic year, while participants in Experiment 2 participated at the beginning of another academic year. The gender distribution was comparable in both experiments, χ^2^(1) = .432, *p* = .51, *V* = .057.

We performed a sample size power analysis (Mayr, Buchner, Erdfelder, & Faul, 2007) for a 2 × 12 mixed-design analysis of variance (ANOVA) based on the number of emotions (*broadness*). This analysis suggested that at an alpha level of .050 and a beta level of .950, and assuming a correlation between repeated measures of .5 and epsilon of 1, the total sample size of 54 is sufficient to detect a medium effect size of .25. We set 27 (i.e., 54/2) as a minimum number of participants for each colour presentation condition and recruited participants over 2 months (April–May) for Experiment 1, and over 2 months (October–November) for Experiment 2. We included all participants who volunteered in this time window (total *N* = 173; *n* = 98 in Experiment 1, *n* = 75 in Experiment 2).

We subsequently excluded participants in case of self-reported (*n* = 3 in Experiment 1) or tested (*n* = 1 in Experiment 2; Ishihara, 1993) colour blindness or who were not native French speakers (*n* = 13 in Experiment 1; *n* = 20 in Experiment 2). We recruited participants from the same student pool but coming from different academic years. Consequently, participants’ age, gender, the level of education, and the native language were matched between the two experiments. Finally, as the data for Experiment 1 were Internet-based, we excluded participants who were too quick or too slow (i.e., took ≤3 or ≥90 minutes; *n* = 4) or did not show minimal engagement with the experiment (i.e., spent ≤20 seconds on the first four colour terms; *n* = 0; see also, Jonauskaite, Dael, et al., 2019).

Participation was voluntary and was rewarded with course credit. Both experiments were conducted in accordance with the principles expressed in the Declaration of Helsinki (World Medical Association, 2013). No specific ethical clearance was received for this study, as the law of Canton of Vaud, Switzerland did not require it.

### Colour Stimuli

We used 12 colour stimuli—*red*, *orange*, *yellow*, *green*, *turquoise*, *blue*, *purple*, *pink*, *brown*, *white*, *grey*, and *black*—labelling the principal colour categories (Biggam, 2012). They were presented as colour terms written in black ink in French (Experiment 1) or as colour patches (Experiment 2). *Turquoise* covers the blue–green range and the remaining 11 stimuli represent French basic colour categories (Morgan, 1993; Table S1). For patches, we displayed the best exemplars of each colour category (i.e., focal colours, Table 1; Lindsey & Brown, 2014), which are largely universally recognised (Regier, Kay, & Cook, 2005).

**Table 1.** Colour Stimuli Used in Experiment 1 (Colour Terms) and Experiment 2 (Colour Patches).

Colour term (Experiment 1)	Colour patch (Experiment 2)					
	Munsell colour order system			CIE1931 coordinates		
	Hue	Value	Chroma	*Y* (cd/m^2^)	*x*	*y*
Red	5.00 R	4	14	12.00	.57	.31
Orange	5.00 YR	6	12	30.05	.51	.42
Yellow	5.00 Y	8	14	59.44	.45	.48
Green	2.50 G	5	12	20.99	.27	.50
Turquoise	7.50 BG	6	8	30.38	.22	.33
Blue	10.00 B	6	10	30.05	.20	.24
Purple	7.50 P	4	10	12.00	.31	.22
Pink	7.50 RP	7	8	43.07	.37	.31
Brown	7.50 YR	3	6	6.55	.49	.42
White	10.00 RP	9.5	0	90.01	.31	.33
Grey	10.00 RP	6	0	30.05	.31	.33
Black	10.00 RP	1.5	0	2.02	.31	.33
Grey (background)	10.00 RP	5	0	18.58	.31	.32

*Note.* Munsell values for colour patches taken from Lindsey and Brown (2014). The last columns show the CIE1931 *xyY* values for our patches.

### Emotion Assessment

**Figure 1.**
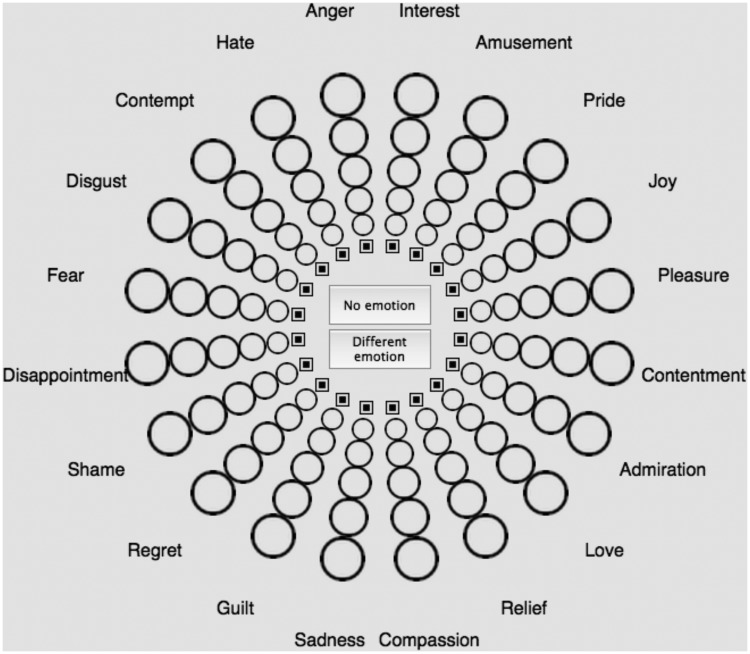
The Geneva Emotion Wheel (GEW) as it appears on the online study (Experiment 1; http://www2.unil.ch/onlinepsylab/colour/main.php). GEW was used in both studies to associate emotion with colour terms or patches and to evaluate the intensity of associated emotions with circles of increasing size (Scherer, 2005; Scherer et al., 2013).

The GEW 3.0 (Figure 1; Scherer, 2005; Scherer et al., 2013) is a self-report measure of the feeling component of emotion. Twenty emotion concepts (Table S2) are represented along the circumference of a wheel, organised around two axes—*valence*, also known as evaluation or pleasantness (horizontal: positive vs. negative), and *power*, also known as control, dominance, or potency (vertical: strong vs. weak). Emotion concepts similar in valence and power are placed close to each other on the GEW. Emotion concepts can further be categorised in terms of *arousal*, also known as activation (high arousal vs. low arousal) based on complementary research studies (Fontaine, 2013; Soriano et al., 2013). Circles of increasing size connect the centre of the wheel with the circumference of the wheel. These circles denote five degrees of emotion intensity, coded from 1 (*smallest circle; weakest intensity*) to 5 (*biggest circle; strongest intensity*), or 0 if no emotion is chosen (*little square*). The Swiss Centre for Affective Sciences provides the validated French version of the GEW (Table S1).

### Procedure: Experiment 1 (Colour Terms)

**Figure 2.**
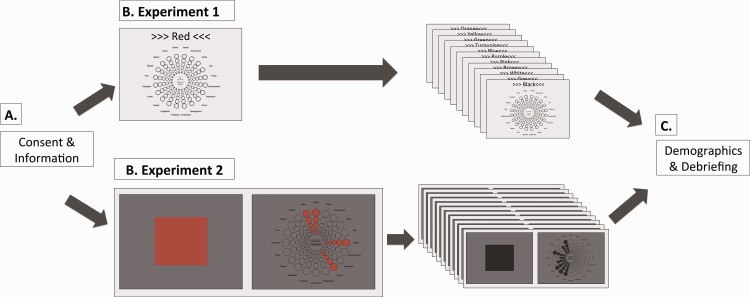
Procedure for Experiments 1 and 2. (a) Participants received written study information and signed informed consent. (b) Main experiment. In Experiment 1, participants saw 12 colour terms in randomised order. They associated colour terms with one, several, or none of the GEW emotion concepts (see “Emotion Assessment” section and Figure 1 for enlarged GEW). In Experiment 2, participants saw 12 colour patches in randomised order. They associated colour patches with one, several, or none of the GEW emotion concepts on the subsequent screen. Here, they saw the small GEW squares as well as the GEW rays of chosen emotion concepts presented in the colour they were currently evaluating. (c) Participants answered demographic questions and were debriefed.

Data for Experiment 1 were collected online from the first-year psychology student pool of the local university. The experiment started with an information page. Participants were informed that they expressed their consent to participate by continuing to the next page. Then, participants were explained the task and performed a manipulation check exercise indicating that they understood the task. Participants were asked to correct the responses of an imaginary person (Peter) and were given feedback. During the experiment, participants saw the 12 colour terms written in black ink (Table 1) presented sequentially and in random order above the GEW on a neutral grey background (see Figure 2). Participants associated one, several, or none of the GEW emotion concepts with each colour term and rated the respective emotion intensity by choosing circles of different sizes (later converted to 1–5 ratings). After the main experiment, participants provided demographic information and were debriefed. They also saw results from a previous related marketing experiment in a graphic form. Participation took on average 12.3 minutes.

We collected the current data as part of a larger on-going International Colour-Emotion Survey online (https://www2.unil.ch/onlinepsylab/colour/main.php; Mohr, Jonauskaite, Dan-Glauser, Uusküla, & Dael, 2018). Given the continuous nature of the survey, we reported part of these data in our study on gendered colours (Jonauskaite, Dael, et al., 2019, Study 3).

### Procedure: Experiment 2 (Colour Patches)

Data for Experiment 2 were collected in the laboratory from the first-year psychology student pool. Upon arrival, participants signed a written informed consent form. We next used a paper version of Ishihara (1993) colour blindness test to assess participants’ colour vision. Afterwards, participants were invited to individual testing rooms, which were dark and illuminated only by a computer monitor. Participants were presented with a colour patch (15° × 15° viewing angle) on a neutral grey background (see Table 1) for a minimum of 5 seconds and instructed to focus on the colour patch. Participants chose when to move to the subsequent page. In analogy to Experiment 1, they associated one, several, or none of the GEW concepts with the target colour patch and rated the intensity of each associated emotion concept. While associating emotions, participants could see the target colour on small GEW squares as well as on the chosen intensity circles (Figure 2(b); Experiment 2). There were three colour patches as practice trials at the beginning of the experiment, with 12 total experimental colour patches presented in randomised order (Table 1, values adapted for each monitor, see “Apparatus” section). We collected these data in the laboratory to ensure accurate colour presentation. Experimenters were available for questions at any point during the experiment. After the main experiment, participants provided demographic information on a paper questionnaire and completed another unrelated experiment, not reported here (choosing focal colours). Finally, they were debriefed. The experiment took approximately 15 minutes (Figure 2).

### Apparatus

The task was performed on four similar monitors (Colour Edge CG243W 24.1″ Widescreen LCD display), which were linearised with an in-built sequence before each session. We used the Konica Minolta CS-100A Chroma Meter to measure the parameters of red, green, and blue guns of each monitor. We report here the white points of the monitors in Commission Internationale de l’Eclairage [CIE] *xyY* colour space (Monitor 1: .318, .334, 73; Monitor 2: .325, .340, 113; Monitor 3: .321, .336, 109; Monitor 4: .318, .323, 128). Although the four monitors had substantially different luminance values for the white points, these differences should not affect our study given that colour presentation was calculated for each monitor separately. Monitor 1 could not produce a luminance value large enough to represent the white patch (see Table 1) and thus it displayed a slightly darker version. Observers’ eyes would always adapt to this maximum value and thus they did not perceive any difference. The gamma curves were estimated from luminance increments of each of the three guns using a standard protocol (Brainard, Pelli, & Robson, 2002). The measured primaries in CIE *xyY* of Monitor 1 were red (*R*) = (.697, .300, 18.9), green (*G*) = (.189, .698, 50.9), and blue (*B*) = (.141, .026, 2.13), Monitor 2—*R* = (.696, .301, 29.6), *G* = (.192, .701, 78.9), and *B* = (.141, .026, 3.17), Monitor 3—*R* = (.695, .301, 28.7), *G* = (0.191, 0.699, 76.8), and *B* = (.141, .028, 3.30), and Monitor 4—*R* = (.696, .301, 34.9), *G* = (.188, .694, 89.5), and *B* = (.141, .027, 4.25). These measurements were then used to convert colour values from monitor-independent *xyY* system to monitor-dependent RGB system and display them in each screen. This step was necessary in order to keep the photometric characteristics of colour stimuli constant across monitors. Viewing was unrestrained and the viewing distance was approximately 70 cm.

### Design and Data Analysis

We employed a mixed design to establish which emotions were associated with which colours (terms and patches together; within-subjects design) and to compare the emotion associations between colour terms and patches (between-subjects design). The independent variables were (a) colour presentation mode (between-subjects; colour term or colour patch), (b) colour (within-subjects, 12 levels, see “Colour Stimuli” section), and (c) emotion (within-subjects, 20 levels, see “Emotion Assessment” section) or emotion dimensions of valence, arousal, and power (within-subjects, 2 levels, see later).

#### Specific emotions

We started the analyses by investigating the specific emotion concepts associated with colours. We calculated the proportion of participants who associated a specific colour with a specific emotion concept by dividing the number of participants who chose each emotion concept for each colour by the total number of participants. The proportion of participants was calculated for each colour presentation condition separately, as well as for both conditions together. The proportion was the first dependent variable (DV1). DV1 varied from 0 (*very unlikely association, no one chose it*) to 1 (*very likely association, everyone chose it*).

We identified the most and least prominent colour–emotion associations with a two-step autocluster analysis using Schwarz’s Bayesian Criterion (Bacher, Wenzig, & Vogler, 2004) on the proportion values of terms and patches together (DV1). To compare the *pattern* of emotion associations, we created two 12 × 20 (Colours × Emotions) representation matrices using the proportion values. Then, we employed representation similarity analyses based on Pearson matrix-to-matrix correlations (Kriegeskorte, 2008) to compare the colour term matrix (12 × 20) with the colour patch matrix (12 × 20), also known as a Pattern Similarity Index (PSI). PSI reflects the degree of similarity in the pattern of colour–emotion associations across terms and patches. A PSI score of 1 indicates perfect pattern similarity, and a PSI score of 0 indicates complete pattern dissimilarity. Furthermore, to compare the similarity of emotion associations between terms and patches for each colour, we calculated PSI_colour_. PSI_colour_ was estimated per colour using Pearson correlations (12 correlations, each time 1 × 20 colour term vector correlated to 1 × 20 colour patch vector).

To test whether the *proportion* of participants endorsing an association differed between terms and patches, we used a mixed-design 2 × 240 ANOVA on proportion values, which accounted for the repeated-measures design. This test indicated whether emotion associations were overall more likely for terms or patches. To identify the source of any dissimilarity, we used Fisher’s (1922) exact tests to compare the proportion of participants endorsing a particular colour–emotion association (yes/no; *n* = 240) for terms and patches.

#### Emotion dimensions

We derived emotion dimensions associated with colours from the number of emotion concepts associated with each colour. For each colour, we counted how many positive and negative (*valence*), high and low arousal (*arousal*), and strong and weak (*power*) emotion concepts each participant chose (Table S2). The number of emotions indicated the *broadness* of associations and was our second dependent variable (DV2). DV2 varied from 0 to 10 for each level of valence, arousal, and power. We conducted a mixed-design 2 × 2 × 12 multivariate analysis of variance (MANOVA) model with broadness of valence, arousal, and power as dependent variables, and levels of emotion dimensions (positive-negative, high-low arousal, and strong-weak), colour presentation mode, and colour as independent variables.

#### Emotion intensity

The third dependent variable (DV3)—*emotion intensity*—was calculated by averaging intensity ratings assigned to emotion concepts associated with each colour. Emotion intensity varied from 1 (*weak*) to 5 (*strong*), unless no emotion concept was chosen (coded as missing value). A series of independent-samples *t* tests compared *emotion intensity* ratings (DV3) between terms and patches overall, and for each colour separately. We used *t* tests to preserve statistical power due to missing values.

Across the statistical tests, where appropriate, we controlled familywise error (type I error) using false discovery rate (FDR) correction and marked the corrected *p* values as *p*_FDR_ (Benjamini & Hochberg, 1995). Alpha level was set at .05; all analyses were two-tailed. We performed analyses and created graphs with the R v.3.4.0 and SPSS v.25.

## Results

### Specific Emotions

**Figure 3.**
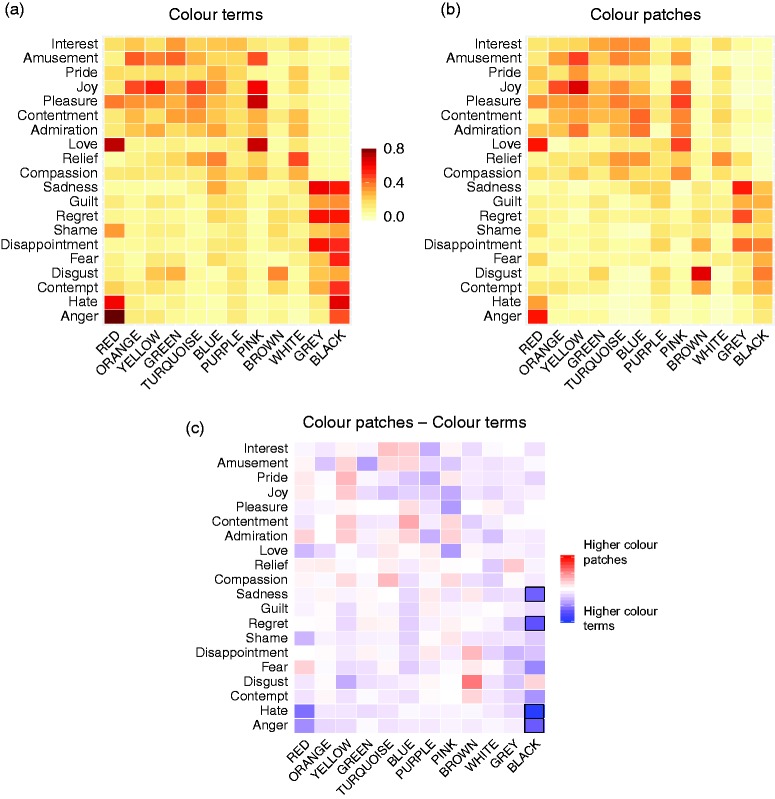
Heatmaps of colour–emotion associations with (a) colour terms, (b) colour patches, and (c) the difference in colour–emotion associations between colour patches and colour terms. (a and b) Redder cells symbolise higher proportions of participants endorsing colour–emotion associations. (c) Redder cells symbolise higher proportions of participants endorsing colour–emotion associations with colour patches; bluer cells symbolise higher proportions of participants endorsing colour–emotion associations with colour terms; dark cell borders indicate statistically significant differences (see *black*).

Across terms and patches, the cluster analysis indicated three clusters of specific colour–emotion associations with a satisfactory goodness of fit (silhouette measure of cohesion and separation was 0.7, Bayesian Information Criterion = 64.9). Cluster 1 included prominent associations (*n* = 26), endorsed by many participants (38%–73%). The most prominent specific colour–emotion associations were *red-anger* (73%), *red-love* (68%), *red-hate* (51%), *red-pleasure* (39%), *orange-joy* (48%), *orange-amusement* (41%), *yellow-joy* (61%), *yellow-amusement* (44%), *turquoise-joy* (45%), *turquoise-pleasure* (41%), *blue-relief* (38%), *pink-pleasure* (63%), *pink-love* (63%), *pink-joy* (55%), *pink-amusement* (42%), *brown-disgust* (50%), *white-relief* (44%), *grey-sadness* (61%), *grey-regret* (55%), *grey-disappointment* (54%), *black-disappointment* (48%), *black-hate* (47%), *black-sadness* (45%), *black-regret* (45%), *black-fear* (45%), and *black-contempt* (43%). *Green* and *purple* had no specific emotion associations in the cluster of the most prominent associations. Cluster 2 included occasional associations (*n* = 77) endorsed by 17% to 37% of participants (e.g., *blue-sadness*, *green-amusement*). Cluster 3 included rare associations (*n* = 137) endorsed by 2% to 17% of participants (e.g., *pink-pride*, *blue-love*, *red-amusement*). We show the colour–emotion associations as proportions in Table S3 for terms and patches together, in Table S4 for terms, in Table S5 for patches, and graphically in Figure 3.

After having established the specific colour–emotion associations, we compared the associations between terms and patches. The PSI comparing the patterns of colour–emotion associations with matrix-to-matrix correlations indicated a high degree of similarity (*r* = .82, *R*^2^ = .672, *p* < .001) between terms and patches (Figure 3(a): terms, Figure 3(b): patches). This result means that similar emotion concepts were associated with colours as terms and as patches. Furthermore, colour-specific PSI_colour_ were high (*r* = .79–.96, *R*^2^ = .624–.922) for all colours except *purple* (*r =* −.23, *R*^2^ = .053, *p*_FDR_ = .340, see Table 2), indicating that the similarity between terms and patches held across all colours except *purple*. The specific emotion concept associations with *purple* as a term were unrelated to those with *purple* as a patch.

**Table 2.** The PSI_colour_ Indicates the Similarity of Colour–Emotion Association Patterns Between Colour Terms and Colour Patches, for Each Colour Separately.

Colour	PSI
Red	.92***
Orange	.94***
Yellow	.90***
Green	.88***
Turquoise	.88***
Blue	.79***
Purple	−.23
Pink	.92***
Brown	.88***
White	.91***
Grey	.96***
Black	.81***

*Note.* All *p* values are FDR corrected for multiple comparisons. PSI = Pattern Similarity Index

***p < .001, PSI = 1 suggests perfect similarity.

When looking at the proportion of participants endorsing colour–emotion associations, the mixed-design ANOVA on the proportion values showed a main effect of presentation mode, *F*(1, 239) = 33.05, *p* < .001, ${\text{η}}_{\text{p}}^{\text{2}}$ = .121. A higher proportion of participants endorsed emotion associations with colour terms than with colour patches. Fisher’s exact tests identified that the associations between *black* and *hate*, *p*_FDR_ < .001, odds ratio [*OR*] = 8.64, 95% CI = [3.59, 22.48]; *black* and *anger*, *p*_FDR_ = .007, *OR* = 5.13, 95% CI = [2.05, 14.26]; *black* and *regret*, *p*_FDR_ = .007, *OR* = 4.48, 95% CI = [1.98, 10.66]; and *black* and *sadness*, *p*_FDR_ = .022, *OR* = 3.85, 95% CI = [1.72, 9.00] were more often chosen when black was a term than a patch. No other comparisons were significant (see Figure 3(c)).

### Emotion Dimensions

The mixed-design MANOVA estimating the number of emotion concepts (broadness) was overall significant; Pillai’s trace value = .55, *F*(1, 130) = 160.0, *p* < .001, ${\text{η}}_{\text{p}}^{\text{2}}$ = .552. The MANOVA indicated a main effect of colour, Pillai’s trace value = .52, *F*(11, 120) = 11.70, *p* < .001, ${\text{η}}_{\text{p}}^{\text{2}}$ = .517, demonstrating that the number of associated emotion concepts varied by colour. This effect was significant for individual mixed-design ANOVAs on valence, *F*(11, 1430) = 15.62, *p* < .001, ${\text{η}}_{\text{p}}^{\text{2}}$ = .107, arousal, *F*(11, 1430) = 15.62, *p* < .001, ${\text{η}}_{\text{p}}^{\text{2}}$ = .107, and power, *F*(11, 1430) = 15.62, *p* < .001, ${\text{η}}_{\text{p}}^{\text{2}}$ = .107. Planned deviation contrasts indicated that *red*, *yellow*, *pink*, and *black* yielded a greater number and *purple*, *brown*, and *white* yielded a smaller number of emotion concepts than expected on average (*p*s ≤ .012; Table 3).

**Table 3.** The Number of Emotion Concepts (Broadness) Associated With Colour Terms and Colour Patches Together and Separately.

Colour	Colour (term and patch)			Colour term			Colour patch			*t* Value	Cohen’s *d*
	*M*	95% CI	Range	*M*	95% CI	Range	*M*	95% CI	Range		
Red	4.70	[3.98, 5.43]	0–20	5.00	[4.02, 5.98]	1–20	4.28	[3.18, 5.37]	0–17	0.98	0.17
Orange	3.45	[2.86, 4.03]	0–20	3.59	[2.72, 4.46]	0–20	3.24	[2.54, 3.94]	0–9	0.62	0.11
Yellow	3.96	[3.32, 4.61]	0–20	3.97	[3.09, 4.86]	0–20	3.94	[2.99, 4.90]	0–16	0.05	0.01
Green	3.64	[3.01, 4.27]	0–20	3.92	[2.99, 4.86]	0–20	3.24	[2.47, 4.01]	0–11	1.13	0.19
Turquoise	3.48	[2.87, 4.1]	0–20	3.47	[2.60, 4.35]	0–20	3.50	[2.67, 4.33]	0–11	−0.04	0.01
Blue	3.95	[3.33, 4.58]	0–20	4.17	[3.24, 5.10]	0–20	3.65	[2.89, 4.41]	1–11	0.86	0.15
Purple	3.26	[2.65, 3.87]	0–20	3.56	[2.64, 4.48]	0–20	2.81	[2.12, 3.51]	0–10	1.30	0.22
Pink	4.23	[3.6, 4.85]	0–20	4.42	[3.54, 5.30]	0–20	3.94	[3.06, 4.83]	1–12	0.77	0.13
Brown	2.53	[1.9, 3.16]	0–20	2.50	[1.58, 3.42]	0–20	2.57	[1.76, 3.39]	0–14	−0.12	0.02
White	3.01	[2.4, 3.61]	0–20	3.45	[2.52, 4.37]	0–20	2.37	[1.73, 3.01]	0–12	1.91	0.32
Grey	3.70	[3.1, 4.31]	0–20	4.13	[3.22, 5.03]	0–20	3.09	[2.39, 3.80]	0–13	1.80	0.31
Black	4.51	[3.87, 5.14]	0–20	5.56	[4.64, 6.49]	0–20	2.98	[2.34, 3.63]	0–10	4.59***	0.77
Overall	3.70	[3.52, 3.88]	0–20	3.98	[3.72, 4.24]	0–20	3.30	[3.07, 3.53]	0–17	1.04	0.19

*Note.* All *p* values are FDR corrected. CI = confidence interval.

***p_FDR_ < .001.

There was no main effect for colour presentation mode, Pillai’s trace value = .01, *F*(1, 130) = 1.38, *p* = .242, ${\text{η}}_{\text{p}}^{\text{2}}$ = .011. This result means that the same number of emotions, on average, was associated with terms and patches. Nonetheless, there was a significant interaction between colour and colour presentation mode, Pillai’s trace value = .27, *F*(11, 120) = 3.97, *p* < .001, ${\text{η}}_{\text{p}}^{\text{2}}$ = .267. This interaction was present for all three individual mixed-design ANOVAs, namely on valence, *F*(11, 1430) = 5.59, *p* < .001, ${\text{η}}_{\text{p}}^{\text{2}}$ = .041, arousal, *F*(11, 1430) = 5.59, *p* < .001, ${\text{η}}_{\text{p}}^{\text{2}}$ = .041, and power, *F*(11, 1430) = 5.59, *p* < .001, ${\text{η}}_{\text{p}}^{\text{2}}$ = .041. Post hoc comparisons revealed that a greater number of emotion concepts was associated with *black* when presented as a term than as a patch (*p*_FDR_ < .001). The term versus patch comparisons were not significant for other colours (all *ps*_FDR_ ≥ .30; Table 3).

#### Valence

Following the results of the mixed-design MANOVA with a mixed-design ANOVA on valence, there was the main effect of valence, *F*(1, 130) = 70.82, *p* < .001, ${\text{η}}_{\text{p}}^{\text{2}}$ = .353, indicating a positivity bias. Participants overall associated more positive (*M* = 2.10, 95% CI = [1.80, 2.40]) than negative (*M* = 1.54, 95% CI = [1.26, 1.83]) emotion concepts with colours. A significant interaction between valence and colour presentation mode, *F*(1, 130) = 5.40, *p* = .022, ${\text{η}}_{\text{p}}^{\text{2}}$ = .040, indicated that this positivity bias was only present for patches (*p*_FDR_ = .012; terms: *p*_FDR_ = .181).

**Figure 4.**
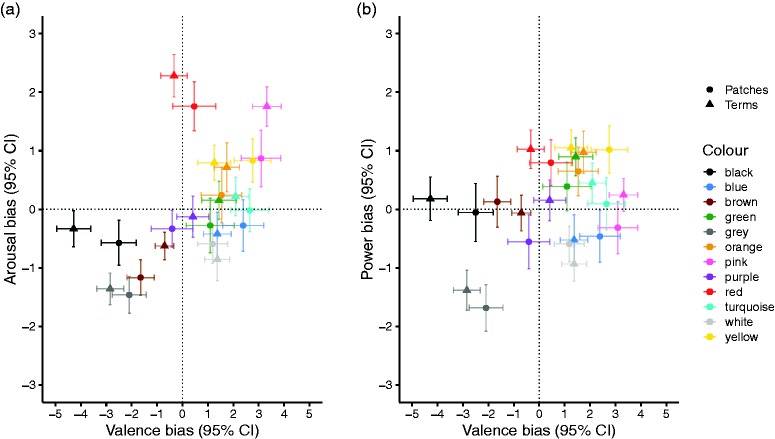
Valence, arousal, and dominance biases of each colour term (triangles) and colour patch (circles). (a) Colours positioned in the Valence × Arousal Space. Valence bias was calculated by subtracting the number of negative emotion concepts from the number of positive emotion concepts associated with each colour (positive–negative); higher values indicate a more positive evaluation. Arousal bias was calculated by subtracting the number of low arousal emotion concepts from the number of high arousal emotion concepts associated with each colour (high arousal–low arousal); higher values indicate a more arousing evaluation. (b) Colours positioned in the Valence × Power Space. Power bias was calculated by subtracting weak emotion concepts from strong emotion concepts associated with each colour (strong–weak); higher values indicate a more empowering evaluation. (a and b) Error bars indicate 95% CIs of the mean. Dotted lines indicate the separation between positive–negative, high arousal–low arousal, and strong–weak emotion concepts. Colours are for visualisation purposes only. See Table S6 for the exact values. CI = confidence interval.

A significant interaction between valence and colour, *F*(11, 1430) = 76.39, *p* < .001, ${\text{η}}_{\text{p}}^{\text{2}}$ = .370, indicated that colours differed in valence. To break down this interaction, we used a series of post hoc *t* tests, FDR adjusted for multiple comparisons, to compare the number of positive and negative emotion concepts associated with each colour (so-called *valence bias*). *Pink*, *white*, *green*, *orange*, *blue*, *yellow*, and *turquoise* were all significantly biased towards positive associations (all *p*s_FDR_ < .001), while *black*, *grey*, and *brown* (all *p*s_FDR_ < .001) were significantly biased towards negative associations. *Red* (*p*_FDR_ = .974) and *purple* (*p*_FDR_ = .765) exhibited no valence bias, meaning that the same number of positive and negative emotion concepts was on average associated with these colours (Figure 4(a) and (b), Table S 6).

A significant three-way interaction between valence, colour, and colour presentation mode, *F*(11, 1430) = 3.70, *p* < .001, ${\text{η}}_{\text{p}}^{\text{2}}$ = .028, suggested that the valence bias varied by colour presentation mode. A series of FDR-corrected *t* tests revealed that a greater number of negative emotion concepts were associated with *black* when presented as a term (*M* = 4.92, 95% CI = [4.26, 5.59]) than as a patch (*M* = 2.74, 95% CI = [2.10, 3.38], *p*_FDR_ < .001). No other comparisons were significant (all *p*s_FDR_ ≥ .085, Figure 4).

#### Arousal

Following the results of the mixed-design MANOVA with a mixed-design ANOVA on arousal, there was no main effect of arousal, *F*(1, 130) = 1.37, *p* = .245, ${\text{η}}_{\text{p}}^{\text{2}}$ = .010. Participants associated the same number of emotion concepts of high (*M* = 1.85, 95% CI = [1.56, 2.14]) and low arousal (*M* = 1.80, 95% CI = [1.51, 2.08]) with colours. A significant two-way interaction between arousal and colour presentation mode was observed, *F*(1, 130) = 9.79, *p* = .002, ${\text{η}}_{\text{p}}^{\text{2}}$ = .070. However, no statistically significant differences were seen in post hoc tests.

A significant two-way interaction between arousal and colour, *F*(11, 1430) = 57.69, *p* < .001, ${\text{η}}_{\text{p}}^{\text{2}}$ = .307, showed that colours differed in how arousing they were (*arousal bias*). FDR-corrected post hoc tests demonstrated that *red* (*p*_FDR_ < .001), *pink* (*p*_FDR_ < .001), *yellow* (*p*_FDR_ < .001), and *orange* (*p*_FDR_ = .001) were associated with a greater number of high compared with low arousal emotion concepts. *Grey* (*p*_FDR_ < .001), *brown* (*p*_FDR_ < .001), *white* (*p*_FDR_ < .001), *black* (*p*_FDR_ < .001), and *blue* (*p*_FDR_ = .012) yielded a greater number of low compared with high arousal emotion concepts. *Purple* (*p*_FDR_ = .084), *turquoise* (*p*_FDR_ = .327), and *green* (*p*_FDR_ = .867) did not differ in terms of arousal (see Figure 4(a); Table S6). The three-way interaction between arousal, colour, and colour presentation mode was not significant, *F*(11, 1430) = 1.63, *p* = .085, ${\text{η}}_{\text{p}}^{\text{2}}$ = .012.

#### Power

Following the results of the mixed-design MANOVA with a mixed-design ANOVA on power, there was no main effect of power, *F*(1, 130) = 2.16, *p* = .144, ${\text{η}}_{\text{p}}^{\text{2}}$ = .016, indicating that participants associated the same number of strong (*M* = 1.85, 95% CI = [1.57, 2.14]) and weak (*M* = 1.79, 95% CI = [1.50, 2.08]) emotion concepts with colours. Despite a two-way interaction effect between power and colour presentation mode, *F*(1, 130) = 7.05, *p* = .009, ${\text{η}}_{\text{p}}^{\text{2}}$ = .051, no statistically significant differences were seen in post hoc tests.

A significant two-way interaction effect between power and colour, *F*(11, 1430) = 31.69, *p* < .001, ${\text{η}}_{\text{p}}^{\text{2}}$ = .196, demonstrated that colours differed in power (*power bias*). FDR-corrected post hoc tests demonstrated that *yellow* (*p*_FDR_ < .001), *red* (*p*_FDR_ < .001), *orange* (*p*_FDR_ < .001), *green* (*p*_FDR_ < .001), and *turquoise* (*p*_FDR_ = .027) were associated with a greater number of strong emotion concepts than weak emotion concepts. *Grey* (*p*_FDR_ < .001), *white* (*p*_FDR_ < .001), and *blue* (*p*_FDR_ = .002) were associated with a greater number of weak compared with strong emotion concepts. *Purple* (*p*_FDR_ = .341), *black* (*p*_FDR_ = .576), *pink* (*p*_FDR_ = .904), and *brown* (*p*_FDR_ = .905) did not differ in terms of power (see Figure 4(b); Table S6). The three-way interaction effect between power, colour, and colour presentation mode was not significant, *F*(11, 1430) = 1.37, *p* = .181, ${\text{η}}_{\text{p}}^{\text{2}}$ = .010.

### Emotion Intensity

An independent samples *t* test showed that participants associated emotion concepts of higher intensity with terms than with patches, *t*(130) = 2.32, *p* = .022, *d* = 0.41. Analyses for individual colours, after the FDR correction for multiple comparisons, indicated that this difference was only present for *black* (Table 4).

**Table 4.** Descriptive Values of the Intensity of the Associated Emotion Concepts With Colour Terms and Colour Patches Together and Separately.

Colour	Colour (term and patch)			Colour term			Colour patch			*N*	*t* value	Cohen’s *d*
	*M*	95% CI	Range	*M*	95% CI	Range	*M*	95% CI	Range			
Red	3.86	[3.70, 4.01]	1.9–5.0	3.99	[3.82, 4.17]	1.9–5.0	3.65	[3.38, 3.93]	1.0–5.0	130	2.16	0.39
Orange	3.32	[3.15, 3.49]	1.0–5.0	3.36	[3.16, 3.57]	1.0–5.0	3.25	[2.95, 3.56]	1.0–5.0	120	0.62	0.11
Yellow	3.55	[3.39, 3.70]	1.0–5.0	3.55	[3.34, 3.76]	1.0–5.0	3.54	[3.30, 3.79]	1.0–5.0	129	0.04	0.01
Green	3.24	[3.07, 3.41]	1.0–5.0	3.38	[3.20, 3.56]	1.0–5.0	3.05	[2.72, 3.38]	1.0–5.0	124	1.90	0.35
Turquoise	3.51	[3.32, 3.71]	1.0–5.0	3.66	[3.42, 3.90]	1.0–5.0	3.31	[2.99, 3.63]	1.0–5.0	118	1.80	0.34
Blue	3.44	[3.29, 3.59]	1.0–5.0	3.49	[3.28, 3.70]	1.0–5.0	3.37	[3.15, 3.60]	1.3–5.0	128	0.74	0.13
Purple	3.21	[3.02, 3.40]	1.0–5.0	3.32	[3.09, 3.55]	1.0–5.0	3.07	[2.75, 3.40]	1.0–5.0	118	1.26	0.23
Pink	3.56	[3.39, 3.74]	1.0–5.0	3.72	[3.50, 3.95]	1.0–5.0	3.34	[3.06, 3.62]	1.0–5.0	129	2.13	0.38
Brown	3.34	[3.14, 3.53]	2.0–5.0	3.33	[3.10, 3.57]	2.0–5.0	3.34	[3.01, 3.68]	1.0–5.0	108	−0.07	−0.01
White	3.56	[3.36, 3.76]	1.7–5.0	3.69	[3.46, 3.92]	1.7–5.0	3.37	[3.01, 3.73]	1.0–5.0	116	1.56	0.30
Grey	3.55	[3.37, 3.72]	1.8–5.0	3.61	[3.38, 3.84]	1.8–5.0	3.45	[3.17, 3.74]	1.0–5.0	128	0.85	0.15
Black	3.62	[3.45, 3.79]	1.8–5.0	3.86	[3.67, 4.04]	1.8–5.0	3.26	[2.95, 3.57]	1.0–5.0	127	3.55**	0.64
Overall	3.49	[3.37, 3.61]	1.8–5.0	3.61	[3.46, 3.75]	1.8–5.0	3.33	[3.12, 3.53]	1.7–4.8	132	2.32*	0.41

*Note.* Significant differences, after the FDR correction, in emotion intensity between colour presentation modes are flagged as *p_FDR_ < .050 and **p_FDR_ < .010. CI = confidence interval.

## Discussion

Research on colour–emotion associations has used colour patches or colour terms. In the former case, the perceptual attributes of colour are considered decisive. In the latter, perceptual attributes are little controlled and linguistic features are considered decisive. In this study, we tested whether colour–emotion associations were comparable when using colour terms and colour patches (similar to Wang et al., 2014). Our French-speaking participants associated a large number of emotion concepts (Scherer, 2005; Scherer et al., 2013) with a representative number of either colour terms or patches and rated the intensity of the associated emotion concepts. In our analyses, we accounted for colour associations with (a) specific emotions, (b) emotion dimensions (valence, arousal, and power; Fontaine et al., 2007), as well as (c) emotion intensity.

Cluster analysis indicated that colour associations with specific emotions differed in terms of frequency. Some associations were frequent (e.g., *red-anger*, *red-love*, *yellow-joy*), other associations occurred occasionally (e.g., *blue-sadness*), and still other associations were rare (e.g., *blue-love*). Some colours were associated with a single specific emotion (*brown-disgust* and *white-relief*), while others with several specific emotions (*red-love*, *red-anger*; *yellow*-*joy*, and *yellow*-*amusement*). Further emotion associations were better described in terms of emotion dimensions. For instance, *black* was associated with mainly *negative* emotions, *grey* with *negative* and *weak* emotions, *blue* with *positive* emotions, and *green* with *positive* and *powerful* emotions. Important to our study, colour associations with specific emotions were very similar (similarity coefficient of .82) when contrasting the associations between colour terms and patches. Similarities ranged from .79 (*blue*) to .96 (*grey*), with the exception of low similarity for *purple* (−.23). The degree of dissimilarity is likely due to additional perceptual or linguistic factors, or perhaps noise in the data. In this study, we observed some systematic dissimilarities between colour terms and colour patches. Participants, overall and particularly for *black*, (a) were more likely to select an emotion concept for terms than for patches and (b) selected emotion concepts of higher intensity for terms than for patches. Participants also associated more negative emotions with *black* when the colour was presented as a term than as a patch.

Our study showed a high degree of similarity in colour associations with specific emotions and emotion dimensions between colour terms and colour patches, at least in a French-speaking population. This similarity might indicate that emotions are associated with an abstract representation of colour (i.e., a colour concept; Abbott et al., 2016). This abstract representation can be accessed via colour perceptions, at least when they are close to focal colours, or basic colour terms. Potentially, similar shades of colour named by the same colour term might be associated with more comparable emotions than similar shades of colour named by different colour terms. For instance, when the colour term *red* denoted both a typical red (i.e., potentially focal) and a dark shade of red, the same emotion associations emerged (i.e., *anger* and *love;* Fugate & Franco, 2019). In contrast, in our study, focal red (i.e., named *red*) was associated with positive and negative emotions (i.e., *love*, *anger*, and *hate*), while light red (i.e., named *pink*) was exclusively associated with positive emotions (i.e., *love*, *joy,* and *pleasure*; see also Fugate & Franco, 2019; Gil & Le Bigot, 2014; Jonauskaite, Dael, et al., 2019; Kaya & Epps, 2004; Sutton & Altarriba, 2016). Despite these observations, lightness and saturation might play a more important role than hue when shades of colour are drastically different from focal colours (e.g., Dael et al., 2016; Palmer et al., 2013; Specker et al., 2018; Valdez & Mehrabian, 1994).

High similarity in emotion associations with colour terms and colour patches held for almost all colours. For instance, we replicated the widely observed association between lightness and positivity (Allan, 2009; Lakens et al., 2013; Meier et al., 2007; Specker et al., 2018; Valdez & Mehrabian, 1994). Whether lightness was perceived or only imagined, *white* was associated with exclusively positive emotions and *black* and *grey* with exclusively negative emotions. Other colours were associated with both known and new emotions. For instance, in addition to associating *yellow* with *joy* (e.g., Fugate & Franco, 2019; Sutton & Altarriba, 2016) and *brown* and *disgust* (e.g., Fugate & Franco, 2019), our participants also associated *yellow* with *amusement*, *orange* with *joy* and *amusement*, and *turquoise* with *joy* and *pleasure*.

The only colour that exhibited different specific emotion associations when presented as a term and as a patch was *purple.* While the pattern of emotion associations with *purple* was uncorrelated between terms and patches, *purple* did not carry widely shared associations with emotion concepts. No emotion concept was chosen for *purple* by more than 20% of participants. Other empirical studies have already suggested that *purple* carries idiosyncratic emotion connotations (Fugate & Franco, 2019; Hemphill, 1996; Hupka et al., 1997; Sutton & Altarriba, 2016). Hence, *purple* might be the most affectively neutral or the most affectively ambiguous colour.

Another colour that was somewhat different between terms and patches was *black*. However, *black* did not differ in terms of *which* emotions were associated, only in terms of *how likely* the associations were. Whether *black* was presented as a term or a patch, it was associated with almost all of the given negative emotions (i.e., *sadness*, *guilt*, *regret*, *disappointment*, *fear*, *disgust*, *contempt*, *hate*, and *anger*; see also Adams & Osgood, 1973; Burkitt & Sheppard, 2014; Fugate & Franco, 2019; Hanada, 2018; Specker et al., 2018; Valdez & Mehrabian, 1994). When *black* was a term, however, the negative associations were stronger and more intense, especially with *hate*, *anger*, *regret*, and *sadness*. Potentially, terms evoke colour percepts that differ from patches. Uusküla and Eessalu (2018) showed that the supposedly *black* glossy Munsell colour chip was named as *black* only by a minority of their participants in a colour naming study. If our *black* colour patch was not *black* enough, participants might have for that reason associated the patch with fewer negative emotions. Another possibility is the difference between *black* in the linguistic and perceptual contexts. *Black* might evoke more negative associations linguistically (e.g., *black magic*, *blackmail*, etc., Allan, 2009) rather than perceptually, the latter triggering notions of sophistication and elegance (Labrecque & Milne, 2012). Hence, while being associated with similar negative emotions perceptually and linguistically, *black* was even more negative in the linguistic context.

Based on different theoretical frameworks, we expected that certain colour–emotion associations might be more prevalent in frequency and intensity when conveyed linguistically (i.e., *blue* and *sadness*, *yellow* and *negative* emotions), while others might be more prevalent in frequency and intensity when conveyed perceptually (i.e., *yellow* and *joy*, *blue* and *positive* emotions). Our data demonstrated that *yellow* was associated with *joy* (see also Jonauskaite, Althaus, et al., 2019; Kaya & Epps, 2004; Sutton & Altarriba, 2016), which is likely explained by its link to sunshine (Jonauskaite, Abdel-Khalek, et al., 2019). *Yellow* was not associated with any *negative* emotion, as some colour expressions would have predicted (e.g., *yellow-bellied* or *rire jaune* [*yellow laughter*]). Our data also showed that *blue* was associated with *positive* emotions (see also Adams & Osgood, 1973; Kaya & Epps, 2004; Manav, 2007; Valdez & Mehrabian, 1994; Wexner, 1954), potentially related to experiences of a clear sky or clean water (Palmer & Schloss, 2010). *Blue* was not in general associated with any *negative* emotion. The only negative association with *blue* was *sadness*, endorsed by 27% of our participants (see also Barchard, Grob, & Roe, 2017; Palmer et al., 2013; Sutton & Altarriba, 2016). This association can be related to colour expressions like *feeling blue* in English and *avoirs des bleus à l’âme* (the soul being bruised, meaning feeling sad, and melancholic) in French. Hence, these examples suggest that—relatively speaking—conceptual colour–emotion associations might have been more strongly reinforced by the perceptual rather than the linguistic systems. A more systematic investigation with a greater number of colours and emotions and a greater number of colour metaphors would be useful to examine this conjecture further.

High emotion similarity between colour terms and colour patches cannot provide equivocal support to the theories favouring the role of language (e.g., conceptual metaphor theory; Lakoff & Johnson, 1999) or perception (e.g., Ecological Valence Theory; Palmer & Schloss, 2010). Of course, these theories have not focussed on colour–emotion associations but instead propose generic association mechanisms. More specific to colour, Wang et al. (2014) proposed a distinction between natural and social colour–emotion associations. Natural associations are believed to be reinforced by perceptual pairings and social associations through linguistic and cultural pairings. They suggested that the association between *red* and *negative* emotions is natural and apparent when *red* is a patch, while an association between *red* and *positive* emotions is social and apparent when *red* is a term. We did not replicate the same distinction in the Swiss French-speaking population neither for *red* nor for any other colour. The valence of all 12 studied colours did not differ between terms and patches. It is unclear to what extent cultural or methodological differences could account for the discrepancy. For instance, in a related study linking 12 colour terms with 20 emotion concepts (Jonauskaite, Wicker, et al., 2019), Chinese participants chose a large number of positive emotions (especially, *love* and *joy*) for the term *red* but also associated *red* with *anger*. Hence, colour–emotion associations might be reinforced by perceptual, linguistic, and cultural systems, and the weight of these factors might vary by culture.

Our results might inform theories of embodied cognition (e.g., Barsalou, 1999), providing evidence for an overlapping representation of linguistic and perceptual stimuli. For instance, the classic Stroop effect (Stroop, 1935) demonstrates how naming the ink of letters is hampered when the letters spell an inconsistent colour term. Similarly, auditory presentation of task-unrelated colour terms has been shown to interfere with discrimination performance for colour patches (Richter & Zwaan, 2009). Linguistic reference to *red*, by including the word *red* in the description of a person, increased their perceived attractiveness (Pazda & Elliot, 2017). Similarly, linguistic reference to *red*, when this word appeared on the exam sheet, hampered students’ intellectual performance (Lichtenfeld, Maier, Elliot, & Pekrun, 2009). Equivalent effects on attractiveness and intellectual performance have been reported for perceptual experiences of *red* (Lehmann, Elliot, & Calin-Jageman, 2018; M. A. Maier, Elliot, & Lichtenfeld, 2008; Mehta & Zhu, 2009). A recent psychophysiological study showed that the words *darkness* and *brightness* triggered comparable pupillary responses to perceptual stimuli (Mathôt, Grainger, & Strijkers, 2017). Imaging studies have also demonstrated shared neural networks of colour perception and colour knowledge in the left fusiform gyrus (Simmons et al., 2007; Slotnick, 2009) and the left lingual gyrus (Hsu, Frankland, & Thompson-Schill, 2012). On the other hand, the existence of distinct neurological conditions such as colour anomia (i.e., inability to name visually presented colours; Davidoff & Ostergaard, 1984), colour agnosia (i.e., inability to recognise colours; Davidoff, 1996), and cerebral achromatopsia (i.e., complete colour blindness after cortical damage; Zeki, 1990) provide evidence for (at least partly) separated neural networks. Hence, it appears that colour perception and colour semantics engage to some degree overlapping neural networks.

In conclusion, our results demonstrate that conceptual associations between emotions and colours were very similar when a colour was presented perceptually (i.e., patch of a focal colour) and linguistically (i.e., a basic colour term). Hence, studies associating emotions with colour terms or patches can be compared and their results integrated (with some caution taken for *purple* and *black*). Future studies can choose to study colour terms or colour patches based on their experimental design and not under the assumption that one method would give more accurate results than the other. Our results would indicate that emotion concepts are associated with a colour concept—an abstract representation of colour—rather than specific perceptual or linguistic properties of colour. This suggestion is true at least when perceived colours relate to focal colours. Future studies may investigate emotion associations with colour patches that are difficult to name (e.g., that border between two neighbouring colour terms; Parraga & Akbarinia, 2016) and with colour patches that vary in lightness and saturation but are named by the same basic term. Our results could also be replicated in a within-subject design to ensure that similarity between colour terms and patches holds within groups. Note that our study cannot determine whether language or perception drives colour–emotion relationships in the first place: We do not know whether colour patches were named or colour terms imagined or both. To disentangle the origins of colour–emotion associations, it would be necessary to move towards less typical populations. For instance, studies in populations that possess different numbers of colour categories would be informative (see M. Maier & Abdel Rahman, 2018). Here, we demonstrated that, once acquired, conceptual colour–emotion associations depend little on how colour is presented.

## Supplemental Material

IPE902484 Supplemental material - Supplemental material for Feeling Blue or Seeing Red? Similar Patterns of Emotion Associations With Colour Patches and Colour Terms Click here for additional data file. Supplemental material, IPE902484 Supplemental material for Feeling Blue or Seeing Red? Similar Patterns of Emotion Associations With Colour Patches and Colour Terms by Domicele Jonauskaite, C. Alejandro Parraga, Michael Quiblier and Christine Mohr in i-Perception

## Note

1 Colour terms in italics (e.g., *yellow*) refer to both a colour patch (i.e., a yellow perceptual stimulus) and a colour term (i.e., *jaune* being yellow in French, because we conducted this study in French).
