# Supplementary material for: Feeling Blue or Seeing Red? Similar Patterns of Emotion Associations With Colour Patches and Colour Terms
Source: Iperception. 2020 Feb 2;11(1):2041669520902484. doi: 10.1177/2041669520902484 (PMC7027086; doi:10.1177/2041669520902484)
Supplement: IPE902484 Supplemental material - Supplemental material for Feeling Blue or Seeing Red? Similar Patterns of Emotion Associations With Colour Patches and Colour Terms [file IPE902484_Supplemental_material.pdf]

Supplementary material

Table S 1. Colour and emotion terms in English and French. The validated French version of emotion terms taken from http://www.affective-sciences.org/gew. French has two basic terms for the "brown" category – *brun* and *marron* (Forbes, 1979). We chose *brun* since it has the least contextually restricted meaning (N. C. W. Spence, 1989). However, we hypothesise that *marron* would result in very similar affective associations to *brun,* since both *brun* and *marron* map to comparable perceptual colours (N. C. W. Spence, 1989).

**English**	**French**
Red	Rouge
Orange	Orange
Yellow	Jaune
Green	Vert
Turquoise	Turquoise
Blue	Bleu
Purple	Violet
Pink	Rose
Brown	Brun
White	Blanc
Grey	Gris
Black	Noir
Interest	Intérêt
Amusement	Amusement
Pride	Fierté
Joy	Joie
Pleasure	Plaisir
Contentment	Contentement
Admiration	Admiration
Love	Amour
Relief	Soulagement
Compassion	Compassion
Sadness	Tristesse
Guilt	Culpabilité
Regret	Regret
Shame	Honte
Disappointment	Déception
Fear	Peur
Disgust	Dégoût
Contempt	Mépris
Hate	Haine
Anger	Colère

Table S 2. The 20 emotions used in the Geneva Emotion Wheel. We show their categorisation regarding on emotion dimensions of valence, arousal, and power (Fontaine, 2013; Scherer, 2005; Scherer et al., 2013; Soriano et al., 2013).

Emotion	Valence	Arousal	Power
Interest	Positive	Low	Strong
Amusement	Positive	High	Strong
Pride	Positive	Low	Strong
Joy	Positive	High	Strong
Pleasure	Positive	High	Strong
Contentment	Positive	Low	Weak
Admiration	Positive	High	Weak
Love	Positive	High	Weak
Relief	Positive	Low	Weak
Compassion	Positive	Low	Weak
Sadness	Negative	Low	Weak
Guilt	Negative	High	Weak
Regret	Negative	Low	Weak
Shame	Negative	High	Weak
Disappointment	Negative	Low	Weak
Fear	Negative	High	Strong
Disgust	Negative	Low	Strong
Contempt	Negative	Low	Strong
Hate	Negative	High	Strong
Anger	Negative	High	Strong

Table S 3. This colour-emotion association matrix indicates the proportion of participants who endorse given colours as being associated with given emotions. These proportions were derived from pooled data, i.e., colour-emotion associations for both colour terms and colour patches.

	BLACK	BLUE	BROWN	GREEN	GREY	ORANGE	PINK	PURPLE	RED	TURQUOISE	WHITE	YELLOW
Interest	.08	.30	.08	.33	.08	.23	.17	.20	.18	.29	.21	.20
Amusement	.03	.26	.05	.36	.03	.41	.42	.18	.13	.33	.08	.44
Pride	.09	.24	.05	.18	.05	.17	.11	.14	.23	.18	.22	.26
Joy	.02	.32	.05	.33	.03	.48	.55	.14	.17	.45	.18	.61
Pleasure	.04	.30	.08	.30	.04	.35	.63	.19	.39	.41	.14	.36
Contentment	.06	.33	.10	.31	.07	.28	.33	.17	.13	.33	.25	.24
Admiration	.05	.36	.07	.19	.06	.26	.33	.12	.20	.27	.23	.36
Love	.03	.12	.06	.11	.04	.07	.63	.17	.68	.11	.15	.10
Relief	.04	.38	.09	.22	.11	.14	.23	.14	.07	.33	.44	.16
Compassion	.05	.25	.10	.14	.08	.16	.32	.20	.17	.20	.25	.19
Sadness	.45	.27	.11	.06	.61	.08	.04	.19	.05	.11	.12	.04
Guilt	.34	.15	.14	.14	.32	.08	.05	.20	.13	.05	.11	.12
Regret	.45	.17	.20	.11	.55	.08	.05	.15	.11	.08	.17	.07
Shame	.27	.06	.18	.11	.22	.08	.08	.16	.28	.04	.08	.11
Disappointment	.48	.12	.20	.09	.54	.08	.05	.19	.08	.07	.11	.08
Fear	.45	.16	.06	.11	.21	.05	.05	.11	.16	.06	.12	.12
Disgust	.35	.02	.50	.27	.20	.08	.06	.19	.11	.05	.04	.18
Contempt	.43	.06	.26	.12	.26	.15	.05	.16	.20	.05	.07	.15
Hate	.47	.03	.07	.08	.11	.11	.04	.13	.51	.04	.03	.05
Anger	.34	.03	.08	.08	.10	.11	.04	.12	.73	.04	.02	.12

Table S 4. This colour-emotion association matrix indicates the proportion of participants who endorse given colours as being associated with given emotions. These proportions were derived from colour-emotion associations for colour terms.

Terms	BLACK	BLUE	BROWN	GREEN	GREY	ORANGE	PINK	PURPLE	RED	TURQUOISE	WHITE	YELLOW
Interest	.10	.26	.12	.35	.08	.26	.15	.27	.19	.23	.22	.19
Amusement	.04	.22	.06	.45	.05	.46	.47	.22	.12	.29	.10	.40
Pride	.13	.29	.06	.19	.06	.18	.09	.22	.21	.21	.24	.19
Joy	.04	.36	.08	.36	.05	.49	.63	.19	.15	.50	.22	.56
Pleasure	.04	.27	.08	.29	.06	.36	.72	.22	.41	.41	.13	.35
Contentment	.06	.26	.14	.33	.06	.28	.29	.19	.15	.35	.27	.19
Admiration	.06	.32	.09	.21	.08	.26	.28	.19	.15	.26	.28	.31
Love	.05	.12	.05	.13	.05	.10	.72	.15	.74	.09	.17	.10
Relief	.05	.40	.09	.22	.06	.12	.23	.13	.05	.31	.49	.17
Compassion	.06	.28	.13	.15	.08	.17	.28	.21	.15	.14	.29	.15
Sadness	.58	.31	.09	.05	.64	.06	.06	.17	.06	.12	.15	.05
Guilt	.37	.19	.15	.13	.33	.08	.06	.18	.14	.06	.10	.15
Regret	.59	.22	.19	.09	.60	.08	.04	.17	.10	.06	.18	.10
Shame	.32	.10	.21	.13	.24	.09	.05	.15	.35	.05	.10	.13
Disappointment	.54	.15	.14	.08	.60	.08	.06	.17	.08	.08	.15	.10
Fear	.55	.21	.04	.14	.26	.05	.05	.13	.12	.05	.12	.15
Disgust	.31	.04	.38	.29	.26	.08	.06	.18	.13	.08	.06	.26
Contempt	.53	.09	.22	.13	.29	.14	.05	.15	.23	.06	.09	.18
Hate	.67	.04	.08	.12	.14	.13	.05	.14	.63	.06	.05	.08
Anger	.47	.05	.10	.09	.12	.14	.05	.14	.83	.06	.03	.15

Table S 5. This colour-emotion association matrix indicates the proportion of participants who endorse given colours as being associated with given emotions. These proportions were derived from colour-emotion associations for colour patches.

Patches	BLACK	BLUE	BROWN	GREEN	GREY	ORANGE	PINK	PURPLE	RED	TURQUOISE	WHITE	YELLOW
Interest	.04	.37	.04	.31	.07	.20	.19	.09	.17	.37	.20	.22
Amusement	.02	.31	.02	.24	.00	.33	.35	.13	.15	.39	.04	.50
Pride	.04	.17	.02	.17	.02	.17	.15	.04	.26	.15	.19	.35
Joy	.00	.26	.02	.28	.00	.48	.44	.07	.20	.37	.13	.69
Pleasure	.04	.35	.07	.30	.00	.33	.50	.15	.37	.41	.17	.37
Contentment	.06	.44	.04	.28	.07	.28	.39	.15	.09	.30	.22	.31
Admiration	.02	.43	.04	.17	.04	.26	.39	.02	.26	.30	.15	.43
Love	.00	.13	.07	.07	.02	.02	.50	.20	.59	.15	.13	.09
Relief	.02	.35	.09	.22	.19	.17	.24	.17	.09	.35	.37	.15
Compassion	.02	.20	.06	.13	.09	.15	.37	.19	.19	.30	.19	.24
Sadness	.26	.22	.15	.07	.57	.09	.00	.22	.04	.11	.07	.02
Guilt	.30	.09	.13	.15	.30	.09	.04	.22	.11	.04	.11	.07
Regret	.24	.11	.20	.13	.48	.09	.07	.13	.11	.09	.15	.02
Shame	.20	.00	.15	.09	.19	.06	.11	.17	.19	.02	.04	.07
Disappointment	.41	.07	.30	.11	.44	.09	.02	.22	.07	.06	.06	.06
Fear	.30	.09	.09	.07	.15	.04	.04	.07	.22	.07	.13	.07
Disgust	.41	.00	.67	.22	.13	.09	.06	.20	.07	.02	.00	.07
Contempt	.30	.02	.31	.11	.20	.17	.06	.17	.17	.02	.04	.11
Hate	.19	.02	.06	.04	.06	.07	.02	.11	.33	.00	.00	.02
Anger	.15	.00	.06	.07	.07	.06	.02	.09	.59	.00	.00	.07

Table S 6. Valence, arousal, and power loadings for each colour, separated by term and patch. The term *Bias* represents the mean loading, and 95% CI represents 95% confidence intervals of the mean. The same information appears in **Error! Reference source not found.**.

**Colour presentation mode**	Colour	**Valence** Bias	*95% CI*	**Arousal** Bias	*95% CI*	**Power** Bias	*95% CI*
Colour term	Red	-0.33	[-0.86, 0.19]	2.28	[1.92, 2.65]	1.03	[0.70, 1.36]
	Orange	1.74	[1.24, 2.24]	0.72	[0.30, 1.14]	0.97	[0.62, 1.33]
	Yellow	1.26	[0.61, 1.91]	0.80	[0.49, 1.10]	1.05	[0.74, 1.36]
	Green	1.44	[0.76, 2.11]	0.15	[-0.17, 0.48]	0.90	[0.58, 1.22]
	Turquoise	2.09	[1.59, 2.61]	0.22	[-0.11, 0.55]	0.45	[0.11, 0.79]
	Blue	1.37	[0.83, 1.92]	-0.42	[-0.80, -0.05]	-0.53	[-0.96, -0.09]
	Purple	0.41	[-0.22, 1.04]	-0.13	[-0.48, 0.22]	0.15	[-0.19, 0.50]
	Pink	3.32	[2.76, 3.89]	1.76	[1.42, 2.09]	0.24	[-0.04, 0.53]
	Brown	-0.71	[-1.07, -0.34]	-0.63	[-0.86, -0.39]	-0.06	[-0.37, 0.24]
	Grey	1.37	[0.88, 1.86]	-0.86	[-1.22, -0.50]	-0.94	[-1.23, -0.64]
	White	-2.85	[-3.37, -2.32]	-1.36	[-1.63, -1.09]	-1.39	[-1.73, -1.04]
	Black	-4.28	[-4.95, -3.61]	-0.33	[-0.64, -0.03]	0.18	[-0.19, 0.55]
Colour patch	Red	0.46	[-0.38, 1.31]	1.76	[1.34, 2.18]	0.80	[0.40, 1.19]
	Orange	1.54	[0.74, 2.33]	0.24	[-0.23, 0.71]	0.65	[0.23, 1.06]
	Yellow	2.76	[2.04, 3.48]	0.83	[0.46, 1.21]	1.02	[0.61, 1.42]
	Green	1.09	[0.15, 2.04]	-0.28	[-0.74, 0.19]	0.39	[-0.02, 0.80]
	Turquoise	2.65	[1.91, 3.39]	-0.02	[-0.39, 0.35]	0.09	[-0.35, 0.54]
	Blue	2.39	[1.59, 3.19]	-0.28	[-0.72, 0.16]	-0.46	[-0.91, -0.02]
	Purple	-0.41	[-1.23, 0.42]	-0.33	[-0.65, -0.02]	-0.56	[-1.02, -0.09]
	Pink	3.09	[2.32, 3.87]	0.87	[0.39, 1.35]	-0.32	[-0.76, 0.13]
	Brown	-1.65	[-2.18, -1.11]	-1.17	[-1.47, -0.86]	0.13	[-0.31, 0.56]
	Grey	1.19	[0.60, 1.78]	-0.59	[-0.92, -0.26]	-0.59	[-0.90, -0.29]
	White	-2.09	[-2.76, -1.43]	-1.46	[-1.78, -1.15]	-1.69	[-2.08, -1.29]
	Black	-2.50	[-3.19, -1.81]	-0.57	[-0.960, -0.19]	-0.06	[-0.55, 0.44]
